# Supplementary material for: Comparative Effectiveness and Safety of Finasteride and Dutasteride in the Treatment of Benign Prostatic Hyperplasia: A Real-World Retrospective Study
Source: Medicina (Kaunas). 2025 Oct 30;61(11):1944. doi: 10.3390/medicina61111944 (PMC12654297; doi:10.3390/medicina61111944)

Figure S1. Comparative Analysis of Groups – Finasteride vs. Dutasteride Before Treatment

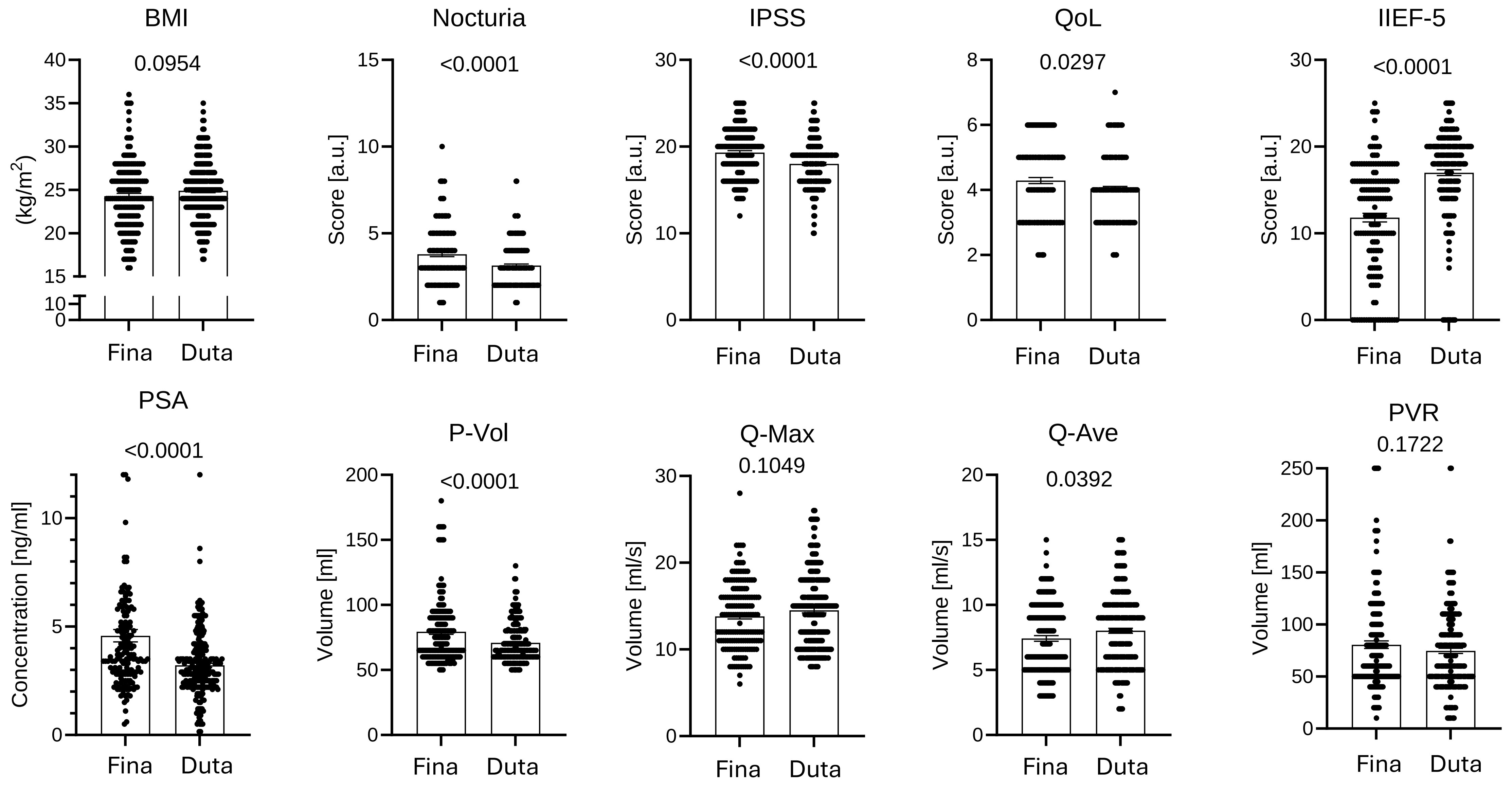

4 data points are outside the axis limits

Figure S2. Overall Analysis – Before and After Treatment

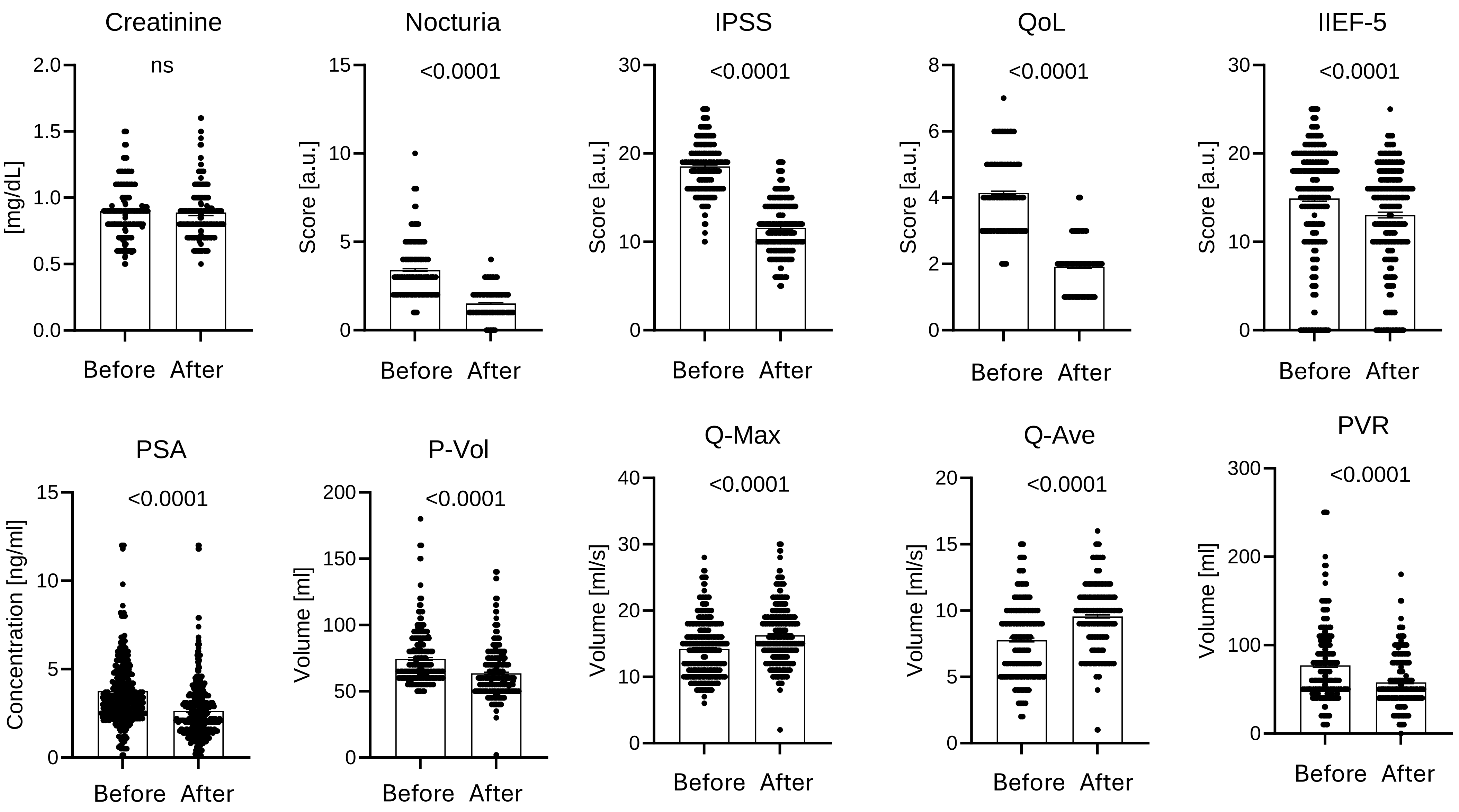

Supplement: Supplementary file 1 [file medicina-61-01944-s001.zip › medicina-3923205-supplementary.pdf]
